# Quantification of lysosphingomyelin and lysosphingomyelin-509 for the screening of acid sphingomyelinase deficiency

**DOI:** 10.1186/s13023-022-02560-x

**Published:** 2022-11-08

**Authors:** Francyne Kubaski, Alberto Burlina, Danilo Pereira, Camilo Silva, Zackary M. Herbst, Franciele B. Trapp, Kristiane Michelin-Tirelli, Franciele F. Lopes, Maira G. Burin, Ana Carolina Brusius-Facchin, Alice B. O. Netto, Edina Poletto, Tamires M. Bernardes, Gerson S. Carvalho, Ney B. Sorte, Fernanda N. Ferreira, Nilza Perin, Marta R. Clivati, Marnie T. S. de Santana, Sandra F. G. Lobos, Emilia K. E. A. Leão, Marcelo P. Coutinho, Paola V. Pinos, Maria L. S. F. Santos, Debora A. Penatti, Charles M. Lourenço, Giulia Polo, Roberto Giugliani

**Affiliations:** 1 Medical Genetics Service, Hospital de Clínicas de Porto Alegre, Porto Alegre, Brazil; 2 PPGMB, UFRGS, Porto Alegre, Brazil; 3 Division of Inherited Metabolic Diseases, Regional Center for Expanded Neontal Screening, Department of Women and Children’s Health, DIDAS Servizi di Diagnostica Integrata, University Hospital Padova, Padua, Italy; 4 Waters Technologies Brazil, São Paulo, Brazil; 5 Innovatox, São Paulo, Brazil; 6 Department of Chemistry, University of Washington, Seattle, USA; 7 Hospital Infantil Sabará, São Paulo, Brazil; 8 Hospital Regional de Caguatinga, Brasilia, Brazil; 9 HUPES, Salvador, Brazil; 10 Private Clinic, São Paulo, Brazil; 11 Hospital Infantil Joana Gusmão, Florianópolis, Brazil; 12 Centro Clivati de Neurologia, Curitiba, Brazil; 13 Clínica de Pediatria e Adolescentes, Salvador, Brazil; 14 Hemocentro da Bahia, Salvador, Brazil; 15 Centro de Referência e Tratamento da Criança, Campos dos Goitacazes, Brazil; 16 Hajar Hospital, Tehran, Iran; 17 Hospital Infantil Pequeno Príncipe, Curitiba, Brazil; 18 Centro Universitário Estácio de Ribeirão Preto, Ribeirão Preto, Brazil; 19 Dasa, São Paulo, Brazil; 20 Casa dos Raros, Porto Alegre, Brazil

**Keywords:** Acid sphingomyelinase deficiency, Lysosphingomyelin, Tandem mass spectrometry, Biomarker, Niemann-Pick type a/b

## Abstract

**Background:**

Acid sphingomyelinase deficiency (ASMD) is a lysosomal disorder caused by deficiency of acid sphingomyelinase (ASM) leading to the accumulation of sphingomyelin (SM) in a variety of cell types. Lysosphingomyelin (LysoSM) is the de-acetylated form of SM and it has been shown as a biomarker for ASMD in tissues, plasma, and dried blood spots (DBS) and lysosphingomyelin-509 (LysoSM509) is the carboxylated analogue of LysoSM. High levels of Lysosphingomyelin 509 (LysoSM509) have also been shown in ASMD patients. In this study, we report the utility of the quantification of LysoSM and LysoSM509 in DBS of patients from Latin America with ASMD by ultra-performance liquid chromatography tandem mass spectrometry (UPLC-MS/MS).

**Results:**

DBS samples from 14 ASMD patients were compared with 15 controls, and 44 general newborns. All patients had their diagnosis confirmed by the quantification of ASM and the measurement of the activity of chitotriosidase. All patients had significantly higher levels of lysoSM and lysoSM509 compared to controls and general newborns.

**Conclusions:**

The quantification of lysosphingolipids in DBS is a valuable tool for the diagnosis of ASMD patients and lysoSM can be useful in the differential diagnosis with NPC. This method is also valuable in the ASMD newborn screening process.

## Introduction

Acid sphingomyelinase deficiency (ASMD) or Niemann-Pick type A/B (OMIM#257,220, and 607,616, respectively) is a lysosomal disorder caused by the deficiency of acid sphingomyelinase (ASM) due to pathogenic variants in the *SMPD1* gene [1–4].

ASM is required for the metabolism of sphingomyelin, and ASMD has a progressive course due to the continuous lysosomal accumulation of sphingomyelin in a variety of cell types. The disease severity is determined by the degree of the organomegaly, presence or absence of neurological impairment, and the rate of progression. There is a broad phenotypical spectrum, with ASMD type A as the most severe form usually presenting infantile neurovisceral impairment (hepatosplenomegaly, neurologic deterioration, failure to thrive, cherry-red spot of the macula of the retina, interstitial lung disease that can lead to infection or respiratory failure) [3, 5, 6]; ASMD type B usually starts later than ASMD type A, with patients showing visceral impairment (hepatosplenomegaly, progressive liver and pulmonary impairment, osteopenia, atherogenic lipid profile) but with no significant neurological impairment [1, 3]; and patients with intermediate symptoms between ASMD type A and ASMD type B are classified as ASMD type A/B and may present some neurological manifestations [3].

ASMD affects all populations with a variable incidence in different ethnicities [7–12]. There is probably underdiagnosis of AMSD, with an incidence estimated at approximately 0.5 per 100,000 live births [2, 13]. The incidence rates are likely to be better defined with the inclusion of lysosomal disorders in newborn screening [14].

Sphingomyelin (SM) is the substrate for ASM that cleaves the phosphorylcholine linkage of SM producing ceramide [15]. SM is a major compound of most cell membranes and coupled with cholesterol constitutes most of the membrane rafts [16–18]. In the deficiency of ASM, there is primary storage of SM, but also secondary storage of other lipids such as cholesterol and gangliosides leading to the impairment of several cellular processes [15, 19].

Lysosphingomyelin (LysoSM), the de-acetylated form of SM and lysosphingomyelin (LysoSM509), the carboxylated analogue of LysoSM, have been shown as a biomarker for ASMD in tissues, plasma, and dried blood spots (DBS) [20–27]. High levels of Lysosphingomyelin 509 (LysoSM509) have also been shown in ASMD patients [23, 28]. Biomarkers such as lysosphingolipids (LysoSM & LysoSM509) can be used as biomarkers for ASMD and Niemann-Pick disease type C (NPC). This quantification is useful for the diagnosis of this patients and these markers can also be used as second-tier in newborn screening [29]. In this study, we report the utility of the quantification of LysoSM and LysoSM509 in DBS for the diagnosis of ASMD, by the investigation of ASMD patients from Latin America.

## Materials and methods

### Samples

Dried blood samples (DBS) were collected from patients at the Medical Genetics Service from Hospital de Clínicas de Porto Alegre (HCPA). All tests were performed as part of the program developed by the LSD Brazil Network, which aims to provide a diagnosis to patients with lysosomal disorders. DBS was collected from 14 ASMD patients and compared with DBS from 15 control samples and 44 general newborns. Plasma was available in 6 samples and leukocytes were separated in 4 of these samples. Ethical approval was obtained from the Ethics Committee of the HCPA (2006–0351). All samples were stored at − 20 °C before the analysis.

### Chemicals and reagents

Ultrapure water was obtained from the Milli-Q system from Millipore (Bedford, USA). Organic solvents such as LC–MS grade methanol, and chloroform were purchased from Sigma Aldrich (Saint Louis, USA), and HPLC grade acetonitrile was purchased from JT Baker® (Radnor, USA). Ultrapure formic acid was purchased from Sigma Aldrich (Saint Louis, USA). As standard Lyso-sphingomyelin-d7 (LysoSM-d7) was purchased from Avanti Polar lipids (Alabaster, USA). The standard stock solution of LysoSM-d7 was prepared with a final concentration of 5 mM (1 mg of LysoSM-d7 was weighed and dissolved in 424 uL of 2:1 chloroform/methanol). The extraction solution with the internal standard was prepared in a solution of 80v/15v/5v (methanol:acetonitrile: water, respectively) at 2.5 nmoL/L and it was stored at − 20 °C [23].

### Sample preparation

#### LysoSM & LysoSM509

Samples were prepared according to the method previously described by Polo et al. [23]. Briefly, a single 3.2 mm disc was punched into a 96-well polypropylene plate with 100uL of 2.5 nmoL/L of the Lyso-SMd7 and the plate was incubated with a shaker (500 RPM) for 1 h. The plate was centrifuged at 3000 G for 5 min. The supernatant was transferred to a new plate and 50uL of MilliQ water was added. 10uL were injected into the ultra-performance liquid chromatography tandem mass spectrometer (UPLC-MS/MS).

#### Enzyme assays

ASM activity was quantified in DBS by MS/MS with the NEOLSD™ from Perkin Elmer (Turku, Finland) [30, 31] and the cutoff was established as below 0.59 nmoL/h/mL. ASM activity was also quantified in DBS or leukocytes by the radioisotopic method with ^14^C by the method described by Pentchev and cols [32]. The cutoff was established as below 4.8 nmoL/24 h/mL in DBS and below 0.74 nmoL/h/mg of protein in leukocytes. The chitotriosidase activity was quantified by fluorimetry in DBS or plasma according to the method described by Hollak and cols [33] and the reference range was from 0 to 44 nmoL/h/mL in DBS and from 8.8 to 132 nmoL/h/mL in plasma.

### UPLC-MS/MS

The mass spectrometer was a Xevo TQ-S micro from Waters (Milford, USA). Separation occurred on an XSelect® CSH™ C18 3.5 µm 2.1 × 50 mm column from Waters (186,005,255) (Milford, USA) that was kept at 55 °C. The method was first developed by Polo et al. [24]. The mobile phase was a gradient elution of 70:30 (water/acetonitrile) with 0.1% formic acid (solution A) to 65:35 (isopropanol/acetonitrile) with 0.1% formic acid. The flow rate was 0.8 mL/min, and the gradient was as follows: at 0 min. 99.5% solution A, 0.75 min. 75% solution A, 1 min. 40% solution A, 1.5 min. 25% solution A, 1.80 min. 0 solution A, 2.15 min. 0 solution A, 2.20 min. 99.5% solution A. The mass spectrometer was operated with electrospray ionization in the positive mode with multiple reaction monitoring (MRM). The MS/MS parameters were: source temperature of 150 °C, capillary of 3.5 kV, cone 30 V, collision energy 22 V, dessolvation temperature 600 °C, dessolvation 1100 L/h, cone 50 L/h. Precursor and product ions (m/z) were used to quantify as follows for LysoSM 465.4, 184; LysoSM-D7 472.4, 184; and LysoSM-509 509,184. 10 uL of each sample was injected with a running time of 2.20 min.

### Statistical analyses

Statistical analyses were performed using GraphPad Prism 8.0. Normality and lognormality tests were performed to verify if the samples were following a normal (Gaussian) distribution by the following methods: Anderson–Darling test, D’Agostino & Pearson test, Shapiro–Wilk test, and Kolmogorov–Smirnov test. The samples were not following a normal distribution, so the Mann–Whitney test was used for the comparison of lysoSM and lysoSM509 levels in untreated ASMD patients, controls, and general newborns, at the level of significance of 0.05. Pearson’s correlation with a 95% confidence interval was used to analyze lysoSM x lysoSM509 and chitotriosidase x lysoSM or LysoSM509 levels.

## Results

### Patient demographics

A total of 14 patients with ASMD, 15 controls, and 44 general newborns were analyzed. All patients with ASMD had ASM deficiency confirmed in leukocytes or DBS by fluorimetry or by MS/MS in DBS (Table 1).

**Table 1:** Distribution of ASMD patients according to age at diagnosis, country, LysoSM, LysoSM509, and ASM activity

ID	Gender	Age at diagnosis	Country	LysoSM (nmoL/L)	LysoSM509 (nmoL/L)	ASM in DBS by MS/MS (nmoL/h/mL)^b^	ASM in DBS or leukocytes by the _14_C method	Chitotriosidase levels (nmoL/h/mL)
1	Female	1.1	Brazil	3356	32,213	0.12	1^c^	1475^e^
2	Male	6.7	Brazil	750	32,767	n/a	0.6^c^	738^e^
3	Female	5.9	Brazil	547	19,826	n/a	0.6^c^	2.3^e^
4	Female	2.8	Brazil	539	22,573	n/a	0.20^d^	944^e^
5	Male	1	Brazil	1490	45,082	n/a	0.10^d^	1334^e^
6^a^	Female	3.9	Brazil	400	21,812	0.05	n/a	n/a
7^a^	Female	12.5	Brazil	361	17,333	0.06	n/a	n/a
8	Female	39.1	Brazil	840	65,404	0.08	n/a	356^f^
9	Male	12.5	Brazil	577	42,215	0.21	n/a	290^f^
10	Female	39.1	Brazil	697	36,031	0.26	1.1^c^	125^f^
11	Male	14.1	Brazil	692	32,365	n/a	0.2^c^	429^e^
12	Male	4.7	Ecuador	277	16,806	0.47	n/a	n/a
13	Female	2.7	Brazil	1244	22,747	0.06	0.7^c^	290^f^
14	Male	0.7	Brazil	790	32,988	n/a	0.5^c^	Undetectable^f^

*n/a* not available, *DBS* dried blood spots, *ASM* acid sphingomyelinase, *ASMD* acid sphingomyelinase deficiency

^a^Patients 6 and 7 are siblings from a non-consanguineous family

^b^ASM cutoff for affected ASMD patients by MS/MS < 0.59 nmoL/h/mL

^c^ASM cutoff for affected ASMD patients in DBS < 4.8 nmoL/24 h/mL by the _14_C radioisotopic method

^d^ASM cutoff for affected ASMD patients in leukocytes < 0.74 nmoL/h/mg of protein by the _14_C radioisotopic method

^e^Reference range of chitotriosidase in plasma = 8.8–132 nmoL/h/mL

^f^Reference range of chitotriosidase in DBS = 0–44 nmoL/h/mL

57% of the patients were females. The mean age for the ASMD patients was 7.9 years of age (range: 7 months to 39.1 years of age). The mean age for the controls was 14 years old (range: 3 months to 69.7 years old).

**Fig. 1:**
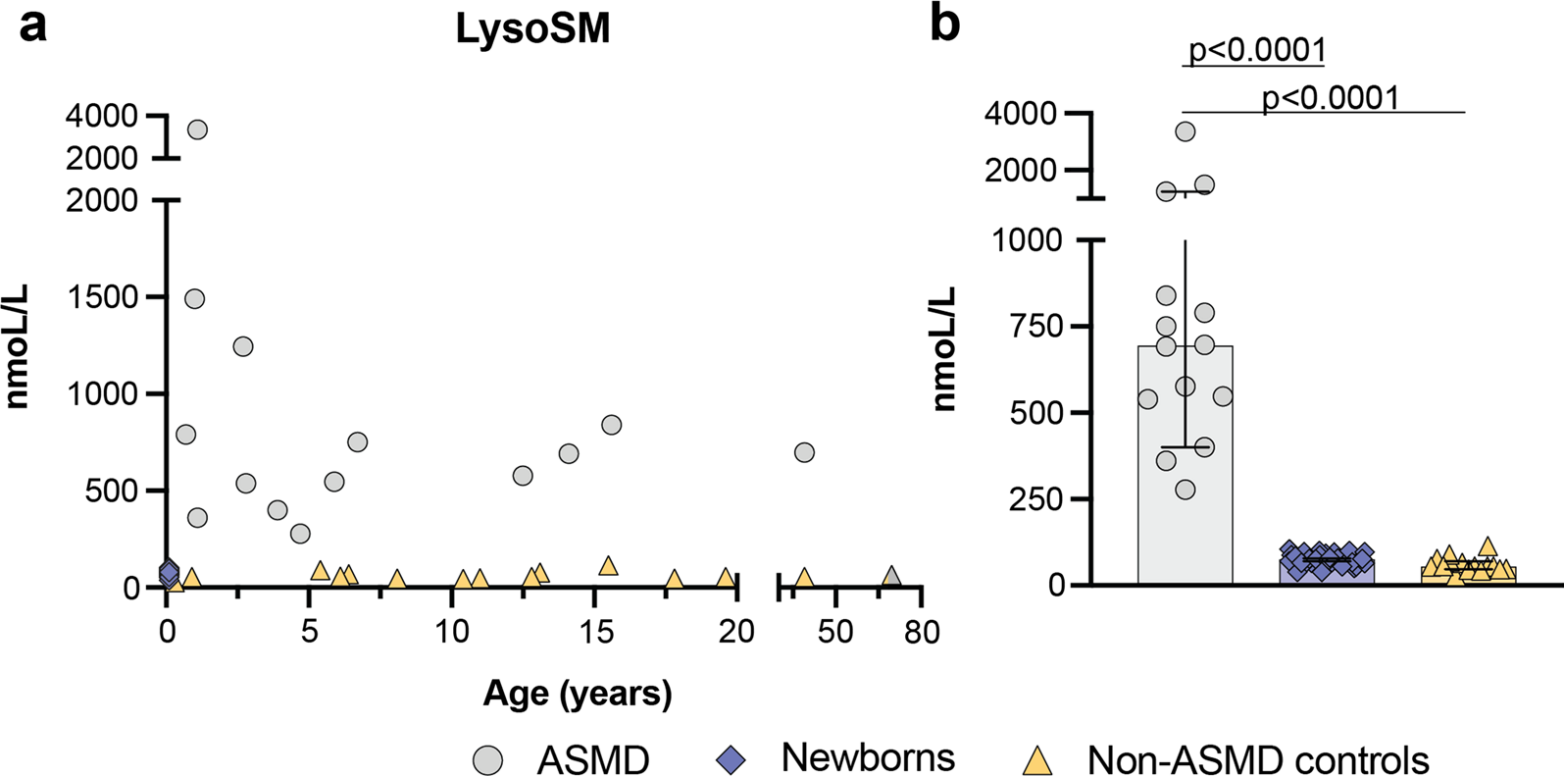
**A** Distribution of lysoSM in ASMD patients, controls, and general newborns according to age. **B** Median levels of lysoSM in ASMD patients, controls, and general newborns with a 95% confidence interval

The mean level of LysoSM in the patient samples was 897 nmoL/L (range: 277–3356 nmoL/L). The mean level of LysoSM in the controls was 60 nmoL/L (range: 28–114 nmoL/L). The mean level of LysoSM in the general newborns was 75 nmoL/L (range: 38–104 nmoL/L) (Fig. 1). The lysoSM levels were significantly higher in ASMD patients compared controls (*P* < 0.0001), and general newborns (*P* < 0.0001) (Fig. 1).

**Fig. 2:**
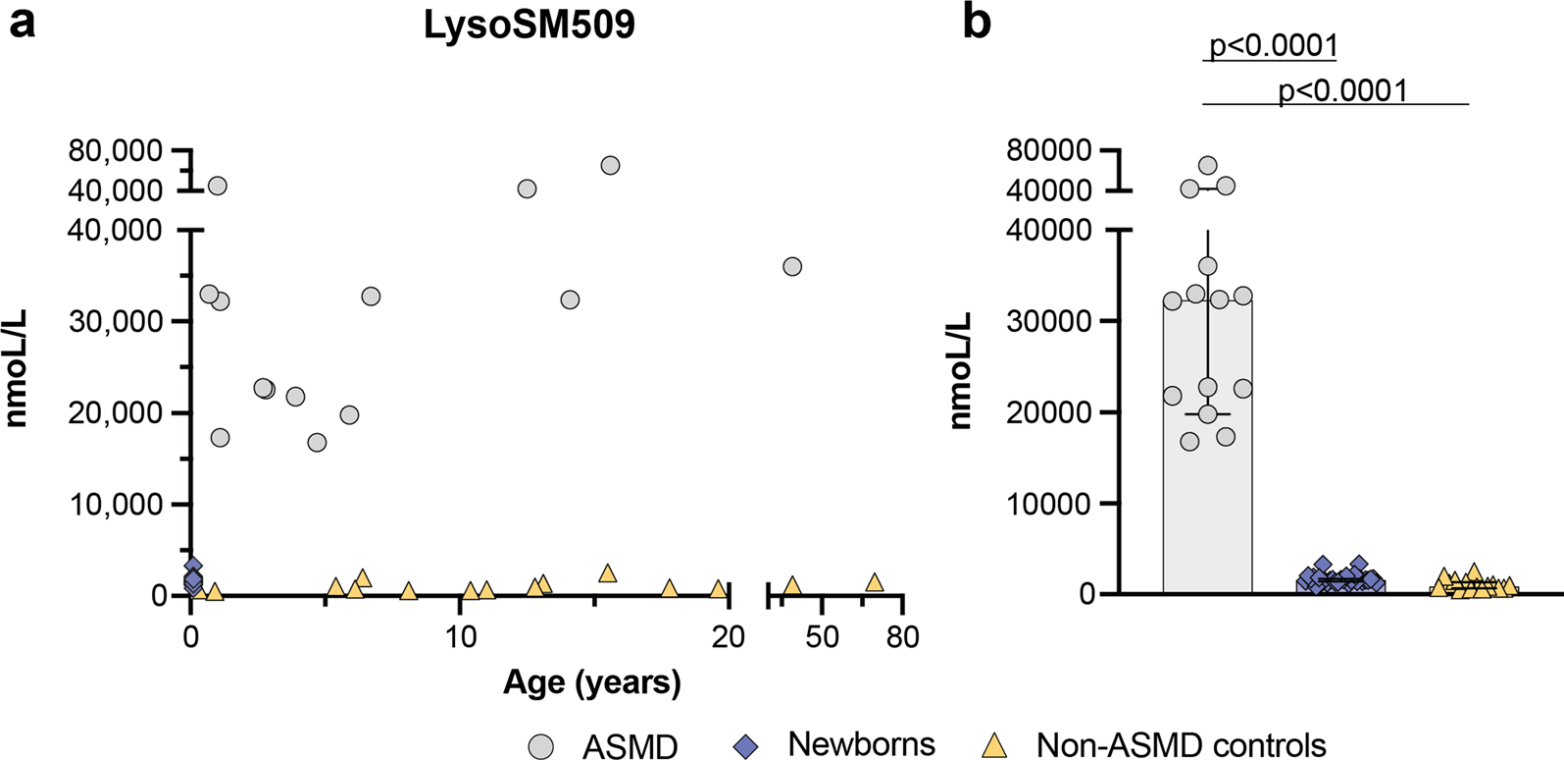
**A** Distribution of lysoSM509 in ASMD patients, controls, and general newborns according to age. **B** Median levels of lysoSM509 in ASMD patients, controls, and general newborns with a 95% confidence interval

The mean level of LysoSM509 in the patient samples was 31,440 nmoL/L (range: 16,806–65,404 nmoL/L). The mean level of LysoSM509 in the controls was 1,088 nmoL/L (range: 536–2,534 nmoL/L). The mean level of LysoSM509 in the general newborns was 1,689 nmoL/L (range: 840–3,332 nmoL/L) (Fig. 2). The lysoSM509 levels were significantly higher in ASMD patients compared to controls (*P* < 0.0001), and general newborns (*P* < 0.0001) (Fig. 2).

**Fig. 3:**
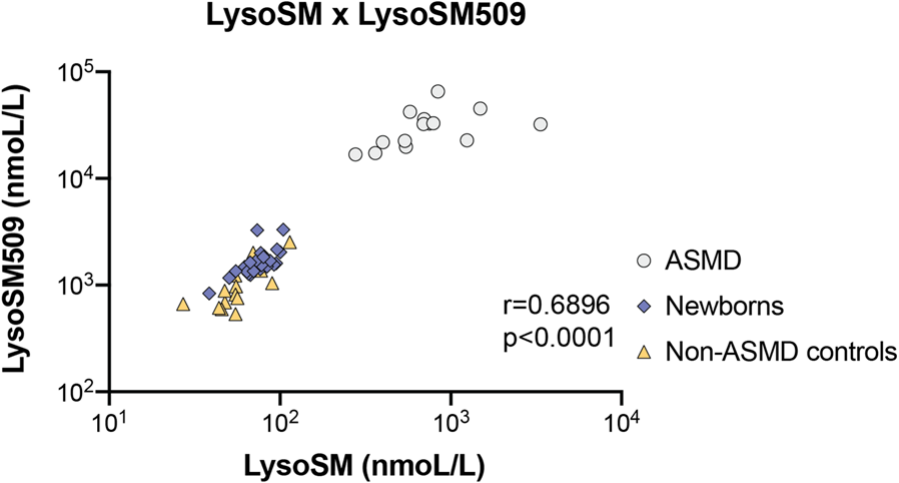
Pearson’s correlation of the lysoSM x lysoSM509 in ASMD patients, controls, and general newborns

There is a positive correlation between the levels of lysoSM and lysoSM509 with Pearson’s correlation coefficient of 0.6896 (*P* < 0001) (Fig. 3). There is no correlation amongst lysoSM *x* age (*P* = 0.4900), and LysoSM509 *x* age (*P* = 0.7151).

Chitotriosidase activity levels were available for 11 patients (Table 1). Five out of 6 samples had elevated levels of chitotriosidase activity in plasma (reference range = 8.8–132 nmoL/h/mL) and one sample had a deficiency of chitotriosidase activity in plasma (Table 1). Four out of 5 samples had elevated levels of chitotriosidase activity in DBS (reference range = 0–44 nmoL/h/mL) and one sample had a deficiency of chitotriosidase activity in DBS (Table 1).

Pearson’s correlation coefficient was used to explore if there was a correlation between the activity levels of plasmatic chitotriosidase x lysoSM or lysoSM509 and the activity levels of chitotriosidase in DBS x lysoSM or lysoSM509. No correlations were observed in the activity levels of plasmatic chitotriosidase, with Pearson’s correlation coefficient of 0.73738 for lysoSM (*P* = 0.0944) and 0.62269 for lysoSM509 (*P* = 0.1867). No correlations were observed in the activity levels of DBS chitotriosidase with Pearson’s correlation coefficient of − 0.25083 for lysoSM (*P* = 0.6316) and − 0.79272 for lysoSM509 (*P* = 0.06).

## Discussion and conclusions

The quantification of lysosphingolipids (lysoSM and lysoSM509) has been shown extremely useful in the diagnosis and monitoring of patients with ASMD and NPC [24, 29, 34–36]. The quantification of lysosphingolipids coupled with chitotriosidase activity has been suggested as a first-tier approach for patients with lipid storage disorders [36].

In this study, we have quantified the levels of lysoSM and lysoSM509 in DBS of patients affected by ASMD, controls, and general newborns. We have seen that lysoSM is a relevant biomarker for the diagnosis of ASMD (Fig. 1, *P* < 0.0001). The levels of lysoSM509 in our patients were very elevated (Fig. 2, *P* < 0.0001) (average = 31,440 nmoL/L, range: 16,806–65,404 nmoL/L) in accordance with data from the literature [22]. The combined determination of both of these biomarkers in a single DBS punch seems to allow the discrimination of ASMD from NPC, as ASMD patients will have elevated levels mainly of lysoSM, while both ASMD and NPC patients will have elevated levels of lysoSM509 [36, 37].

Increased levels of chitotriosidase activity have already been reported elevated in several LSDs due to macrophage activation [1, 33, 36, 38–40]. In our patients, 82% of them had elevated levels of chitotriosidase activity and two had deficient levels that are possibly due to polymorphisms or variants (molecular analysis of the *CHIT1* would be needed to confirm this assumption) (Table 1) [1, 41, 42]. Furthermore, there was no correlation between higher levels of chitotriosidase activity and higher levels of lysoSM & lysoSM509.

In this study, we were limited by the lack of clinical information so we could not correlate the levels of lysosphingolipids with clinical severity. We were also limited by the fact that we did not have information about their genotypes to perform a correlation between the genotype and the lysoSM & lysoSM509 biochemical phenotype. Another limitation was due to the fact that no newborn ASMD samples were available to further evaluate age-correlation. However, our youngest patient was 7 months-old (Table 1).

The fact that both of these lysosphingolipids can be assayed in a single DBS sample makes its measurement very convenient, especially for large countries like Brazil where shipment of samples in refrigerated packs faces many difficulties. This kind of shipment is even more difficult when country borders need to be crossed. In May of 2021, the Brazilian government has approved the inclusion of LSDs in the nationwide public newborn screening program (Law 14.124/2021) [43]. The Brazilian Ministry of Health has not defined which LSDs will be included yet, but ASMD should fulfill criteria to be included, as the activity of ASM and/or the levels of lysoSM and lysoSM509 can be measured in DBS, with genotyping of the *SMPD1* gene also possible in the same DBS sample, allowing diagnosis of the disease in the newborn period, which will be even more important as specific therapies are becoming available.

## Data Availability

The datasets used and/or analysed during the current study are available from the corresponding author on reasonable request.
